# A Narrative Review of Telomere Length Modulation Through Diverse Yoga and Meditation Styles: Current Insights and Prospective Avenues

**DOI:** 10.7759/cureus.46130

**Published:** 2023-09-28

**Authors:** Vahe Aghajanyan, Supriya Bhupathy, Shazia Sheikh, Fauzia Nausheen

**Affiliations:** 1 Medical Education, California University of Science and Medicine, Colton, USA; 2 Education, California University of Science and Medicine, Colton, USA

**Keywords:** telomerase, aging, telomere length, meditation, yoga

## Abstract

Mindfulness practices have demonstrated the potential to positively impact various aspects of human health associated with telomere length (TL) - a recognized marker of healthy aging and susceptibility to age-related diseases. This review seeks to conduct an in-depth comparative analysis, examining methodological variations, outcome assessments, strengths, weaknesses, and gaps across mindfulness-focused studies concerning TL and attrition rates. While emerging data tentatively suggest a positive connection between mindfulness practices and TL, a notable research gap pertains to establishing the clinically recommended dosage of yoga/meditation and mindfulness interventions to effectively influence TL. To address this gap, upcoming research should prioritize meticulous structuring, pedagogical precision, and vigilant monitoring of mindfulness interventions to yield psychological and physiological benefits across an appropriate timeframe and intensity. The amalgamation of yoga/meditation or mindfulness emerges as a promising avenue for enhancing the quality of life while counteracting the influence of telomere attrition in the spectrum of age-related diseases. The core objective of this review is to meticulously investigate the interplay between yoga/meditation and mindfulness practices and their potential impact on TL - an essential biomarker indicative of age-related health and well-being. To achieve this, our study methodically compares various methodological approaches, outcome measures, strengths, and limitations within relevant research endeavors focused on TL and attrition rates. Through this scrutiny, we highlight prevailing research gaps. Our analysis underscores the need for comprehensive research efforts aimed at establishing the optimal therapeutic regimen for yielding significant clinical effects on TL and overall health. In summation, our exploration emphasizes the urgency of further studies to unravel the most effective approaches for positively influencing TL and its implications for holistic health.

## Introduction and background

Biology of telomere and telomerase

Our genetic makeup is housed within chromosomes, composed of DNA and proteins. At the tip of these chromosomes lie telomeres, repetitive sequences of DNA and proteins that play a crucial role in maintaining the integrity of chromosomes. As we age, telomeres gradually shorten, a process referred to as "telomere erosion." These telomeres are vital for preventing the degradation and fusion of chromosome ends during cell division. However, they face vulnerability due to the end replication problem, a consequence of the limitations of DNA polymerase in replicating chromosome ends. When telomeres become critically short, cellular senescence is triggered, hastening the process of cellular aging. This phenomenon is interconnected with aging, age-related diseases, and even cancer. An enzyme called telomerase counters telomere shortening by adding repetitive DNA sequences to chromosome ends during cell division, thus preserving telomere length (TL) and cellular function [1-12]. Telomeres, which serve as protective caps at the ends of chromosomes, play a crucial role in maintaining the stability of the genome and cellular function. Shortened telomeres are associated with aging and age-related diseases. Telomerase, an enzyme, counteracts telomere shortening by adding repetitive DNA sequences, thus influencing cellular lifespan.

Role of telomeres and telomerase in aging and age-related diseases

Telomeres serve a multifaceted role in various cellular processes, including cell senescence, transformation, aging, organ functionality, and cancer. These sequences function by orchestrating cellular responses to stress and growth signals. Factors such as cell division, DNA-damaging stressors like oxidants, and the constrained activity of telomerase contribute to telomere erosion.

Factors that impact TL and telomerase level

Lifestyle components, including psychological stress, anxiety, depression, diet, obesity, smoking, and sleep, can impact telomerase activity and TL. These factors underscore the pivotal role of telomeres in human health and highlight the importance of exploring interventions that could potentially influence their dynamic [13-17].

Yoga and mindfulness-based meditation

Mindfulness is defined as “a focused awareness of one’s experience, and purposeful and nonjudgmental focus on the present moment,” and the versatility inherent in this definition is mirrored in the various approaches employed to attain it. Meditation and yoga are the predominant techniques in contemporary mindfulness programs like mindfulness-based stress reduction (MBSR). These practices have the potential to mitigate stress, anxiety, sadness, and depression [18,19]. In addition to their psychological benefits, yoga can also enhance physical health and quality of life. The reduction of psychological distress through mindfulness practices may potentially impact telomere regulation and length. Mindfulness, meditation, and yoga have garnered growing attention for their potential influence on cellular aging and longevity through their impact on telomeres and telomerase. Recent research has delved into the connection between these practices and the dynamics of telomeres and telomerase, revealing intriguing associations [11,13].

Mindfulness practices, often cultivated through meditation, have been implicated in potentially influencing telomere dynamics. Studies indicate that mindfulness interventions might contribute to telomere maintenance and elongation. MBSR programs have shown links with increased telomerase activity and longer telomeres in leukocytes, suggesting a possible protective effect against cellular aging [20,21]. Similarly, yoga, which encompasses physical postures, controlled breathing, and meditation, has been explored in relation to telomere biology. Certain yoga practices have been associated with higher telomerase activity and longer telomeres in practitioners, indicating a potential role in cellular health and longevity [22,23]. Although limited studies suggest this, rigorous scientific investigations are pivotal to uncover the intricate relationship between these yoga and mindfulness meditation practices on telomeres dynamics.

The science behind yoga, mindfulness, and meditation

Mindfulness, meditation, and yoga are interconnected practices with significant implications for mental and physical well-being. Mindfulness entails the focused awareness of present experiences, fostering the acceptance of thoughts and emotions without judgment. Meditation encompasses diverse techniques aimed at cultivating mindfulness and enhancing concentration. Mindfulness, often realized through meditation, involves directing attention to the present moment without judgment. Meditation practices encompass various techniques such as mindfulness meditation, loving-kindness meditation, and focused attention meditation. These techniques have exhibited positive effects on emotional regulation, attentional control, and stress reduction [24-26]. Similarly, yoga integrates physical postures (asanas) with controlled breathing (pranayama) and meditation to enhance physical flexibility, strength, and mental clarity. Yoga combines physical postures, controlled breathing, and meditation to promote holistic health. These practices have gained attention in contemporary research due to their potential benefits in reducing stress, anxiety, and depression while enhancing the overall quality of life [18,27]. Research suggests that yoga may contribute to improved mood, reduced anxiety, and enhanced well-being through its dual focus on physical and mental dimensions [27,28].

Empirical evidence underscores these practices’ potential to influence psychological and physiological processes. Mindfulness interventions have been associated with alterations in brain activity patterns and structural changes in brain regions linked to emotional processing and self-awareness [26,29]. Similarly, investigations into the effects of meditation and yoga reveal changes in autonomic nervous system activity, cortisol levels, and immune responses, indicative of their impact on stress regulation and overall health [28,30,31].

Impact of mindfulness meditation and yoga on TL

Emerging evidence underscores the potential impact of mindfulness, meditation, and yoga on telomere-telomerase systems. Yoga practices have emerged as potential factors influencing TL and telomerase levels, which are critical markers of cellular aging and health. Research suggests that regular yoga practice may contribute to maintaining TL and enhancing telomerase activity. Several studies have reported positive associations between yoga interventions and telomere dynamics. For instance, Schutze [23] found that yoga practitioners exhibited longer telomeres compared to non-practitioners, indicating a potential role in cellular longevity. Lavretsky [22] observed increased telomerase activity in individuals practicing yoga and meditation, suggesting a mechanism for preserving TL. Additionally, Hoge [11] demonstrated that mindfulness meditation, often integrated into yoga, was associated with reduced telomere attrition, indicating a potential protective effect against cellular aging. Epel [21] reported that MBSR programs were associated with increased telomerase activity and longer telomeres in leukocytes. Other studies, such as Lengacher [32] and Carlson [33], also found positive associations between yoga and meditation interventions and improved TL and telomerase activity. However, variations in study designs, intervention durations, and participant characteristics contribute to the complexity of these findings. While some studies support the link between yoga practices and improved TL and telomerase levels, further well-controlled and longitudinal investigations are necessary to establish a definitive connection.

Our hypothesis posits that existing literature will align with the idea that interventions involving yoga, meditation, and mindfulness contribute to the elongation of telomeres. We anticipate that randomized controlled trials will serve as the optimal methodology for substantiating this hypothesis, given their capacity to mitigate confounding variables and ensure the comparability of study groups.

The primary objective of this systematic review is to comprehensively analyze the methodologies, intervention durations, and types of interventions used in studies exploring the effects of yoga, mindfulness, and meditation on TL. By elucidating these associations, this review aims to provide a foundation for the development of evidence-based research strategies and interventions that investigate the intricate links between mindfulness meditation and yoga, and the intricate dynamics of telomeres and telomerase. Ultimately, this endeavor will contribute to a deeper understanding of the underlying mechanisms and potential clinical implications of these interventions, guiding future investigations in this field.

## Review

Methods and materials

We conducted a comprehensive literature search using electronic databases, including PubMed, Medline, and Google Scholar, and cross-referenced to identify relevant studies for inclusion in this review. Our search strategy combined keywords and Medical Subject Headings (MeSH terms) related to telomere, telomerase, yoga, meditation, and mindfulness. We limited the search to articles published in English, primarily up to date, and also conducted a manual search for relevant review studies.

Our inclusion criteria encompassed observational studies, clinical trials, systematic reviews, and meta-analyses that focused on the impact of yoga, meditation, and mindfulness practices, including their dosage, on telomere and TL. We reviewed the literature for evidence of the impact of mindfulness, yoga, and meditation practices on TL from 2009 to 2023, referencing specific studies [14,15,17].

For each study, we extracted participant numbers, study design, intervention period, and methods used to assess the relationship between yoga, meditation, and mindfulness practices and TL. When reviewing the results and conclusions, we categorized their findings into "significant positive effects," "negligible effects," and "significant negative effects" on TL. Additionally, we provided an assessment of the strengths and weaknesses of each study reviewed.

Intervention duration and telomere effects: Reviewing key studies for future insights

A comprehensive review of 14 studies on the relationship between meditation practices and TL reveals varied outcomes. In a nine-month meditation efficacy trial involving 298 participants, no significant impact on TL was observed, but a notable link emerged between telomere changes and cortical thickness changes in specific brain regions, suggesting a potential mechanical connection between short-term telomere changes and brain structure [34]. In a six-week meditation workshop study with 142 participants, TL decreased in mindfulness meditation and control groups but not in the loving-kindness meditation group [35]. Cross-sectional research involving 17 long-term mindfulness meditators showed reduced age-related TL changes compared to controls, with associations found between TL and DNA methylation patterns [14]. Conversely, a non-interventional study of 15 loving-kindness meditation practitioners and 22 controls revealed longer telomeres in practitioners, particularly among women, even after adjusting for BMI and past depression, although the sample size was relatively small [11]. In a study of 20 Zen meditators and 20 controls, meditators exhibited longer mean TL, with age, experiential avoidance, and self-compassion contributing significantly to TL, though random assignment was not used, and complex processes were measured, introducing potential limitations [15]. A study involving 92 distressed breast cancer survivors found that mindfulness-based cancer recovery and supportive-expressive group therapy maintained TL relative to controls, though the study had limitations in sample size and duration [33]. A study with 62 participants investigated the effects of Insight meditation retreats on TL, observing a short-term increase during the retreat but lacking examination of long-term effects and noting assay variability [13]. A trial with 48 participants explored the impact of transcendental meditation and health education on telomerase gene expression in hypertensive patients, with both groups exhibiting increased expression, but the study had a small sample size and lacked long-term follow-up data [36]. A study with 60 participants examined the effects of Kirtan Kriya Meditation and music listening on cognitive function in individuals with subjective cognitive decline, finding improvements in cognitive function but lacking specific details on assessments and participant characteristics [37]. In a randomized controlled trial with 158 participants, MBSR improved depressive symptoms, mindfulness, and self-compassion but did not significantly impact TL, and the study had a relatively short intervention period and lacked a control group with no intervention [38]. An examination of 33 participants, with 15 yoga practitioners and 18 controls matched for age, gender, and BMI, found that yoga practitioners had longer TL and lower stress markers, suggesting the potential benefits of yoga on cellular aging [39]. A study with 134 breast cancer patients found that a six-week mindfulness program increased telomerase activity but did not significantly affect TL [40]. In a study involving 58 participants with major depressive disorder, a 12-week yoga and meditation intervention aimed to reduce depressive symptoms and assess cellular health biomarkers, though specific findings were not provided [41]. Lastly, a study of 501 participants with psychiatric conditions and healthy controls found shorter TL in psychiatric patients, and an eight-week mindfulness group therapy had no significant impact on TL [42]. These studies collectively highlight the complex and context-dependent relationship between meditation practices and telomere biology, with various factors influencing outcomes across diverse study designs and populations (Table 1, Table 2).

**Table 1:** Reviewing Key Studies: Design, Type of Study and Intervention Duration, and Telomere Effects

No.	Study and Number of Participants	Type of Intervention and Study Design	Period of Intervention	Results and Conclusions
1	Puhlmann et al. [34] (Total: 298)	Open-label efficacy trial of three consecutive 3-month mental training modules with healthy, meditation-naive adults as part of the ReSource Project. Intention-to-treat analysis.	3 consecutive 3-month intervals (9 months total)	- LTL and CT were related to each other. - Mental training did not significantly affect LTL. - Change in LTL was associated with CT changes in specific brain regions. - Mental training did not influence LTL in healthy, middle-aged adults. - Suggests that short-term LTL change may represent transient change.
2	Le Nguyen et al. [35] (Total: 142)	Randomized control trial on learning mindfulness meditation (MM) vs. loving-kindness meditation (LKM) in participants blinded to study hypothesis.	6 weeks (12 weeks total)	- TL decreased significantly in the MM and control groups but not in the LKM group. - MM group showed intermediate TL changes.
3	Mendioroz et al. [14] (Total: 34)	Cross-sectional study comparing long-term mindfulness meditators (MMs) vs. controls.	Long-term Meditators MMs for ten years	- Effects of age on telomere length were drastically reduced in meditators compared to non-meditator controls. - Telomere length was longer in meditators, especially after controlling for age. - Associations found between telomere length and DNA methylation in specific genes.
4	Hoge et al. [11] (Total: 37)	Comparing loving-kindness meditation (LKM) practitioners vs. controls.	4 years of Loving Kindness Meditation LKM	- LKM practitioners had significantly longer relative telomere length (RTL), especially among women. No significant differences among males.
5	Alda et al. [15] (Total: 40)	Comparing Zen meditation experts vs. matched controls.	3 Months Total	- Meditators had significantly longer mean telomere length (MTL) and lower percentage of short telomeres in individual cells than controls. Correlation between telomere length and gene methylation.
6	Carlson et al. [33] (Total: 92)	Randomized control trial comparing mindfulness-based cancer recovery (MBCR) vs. support-expressive group therapy (SET) vs. control.	3 months total	- No statistical difference in post-intervention between MBCR and SET. - Trend toward a difference in T/S ratios between treatment and control. - No associations between changes in stress or mood scores and TL changes.
7	Conklin et al. [13] (Total: 62)	Non-randomized control trial comparing silent meditation retreat participants vs. controls.	3 weeks	- Retreat group showed a short-term increase in telomere length equivalent to the decline typically observed over about 4 years of aging. - No significant difference in telomerase activity between groups.
8	Duraimani et al. [36] (Total: 48)	Randomized single-blind control trial comparing Transcendental Meditation (TM) vs. health education in 48 participants.	16 weeks	- Both TM and health education groups showed increased telomerase gene expression and reduced blood pressure. No significant telomere length changes.
9	Innes et al. [37] (Total: 60)	Randomized control trial comparing Kirtan Kriya (KK) Meditation vs. Music Listening (ML) in participants with Subjective Cognitive Decline (SCD).	3 months	- Both KK and ML groups showed improvements in memory function and cognitive performance at 3 months.
10	Keng et al. [38] (Total: 158)	Randomized control trial comparing Mindfulness-Based Stress Reduction (MBSR) vs. Music-Therapy Based Stress Reduction (MTSR) in participants.	8 weeks	- MBSR group demonstrated greater improvements in depressive symptoms, trait mindfulness, and self-compassion compared to the control. No significant impact on telomere length.
11	Krishna et al. [39] (Total: 33)	Prospective case-control study comparing yoga practitioners vs. control group.	6 weeks	- Yoga practitioners had longer telomere length, lower oxidative stress markers, and lower homocysteine levels compared to controls. Negative correlation between telomere length and systemic stress markers.
12	Lengacher et al. [40] (Total: 134)	Randomized wait-list control study on the effect of Mindfulness-Based Stress Reduction (MBSR) on telomere length (TL) and telomerase activity (TA) in breast cancer patients.	6 weeks	- MBSR led to greater increase in telomerase activity (TA) compared to standard care, but no significant change in TL.
13	Tolahunase et al. [41] (Total: 58)	Randomized control study comparing a yoga and meditation-based lifestyle intervention (YMLI) vs. control in participants with Major Depressive Disorder (MDD).	12 weeks	- Both YMLI and control groups demonstrated increased neuroplasticity biomarkers. No specific impact on telomere length mentioned.
14	Wang et al. [42] (Total: 501)	Single-blind randomized control study comparing mindfulness-based group therapy vs. standard treatment in psychiatric patients.	8 weeks	- Psychiatric patients had shorter telomere length compared to controls. - No significant change in telomere length after 8-week treatment in either.

**Table 2:** Reviewing Key Studies: Methodologies, Strengths, and Weaknesses

No.	Study	Methodology	Study Weaknesses	Study Strengths
1	Puhlmann et al. [34]	- Blood collected at 4 data points and frozen at -80 degrees C. - Genomic DNA extracted from whole blood using QIAamp DNA blood mini kit. - Telomere length measured using quantitative PCR assay and T/S ratios.	- Potential for measurement error in mean LTL T/S ratio. - Not enough participants for adequate statistical power. - Study protocol not preregistered. - Study groups unbalanced with respect to baseline positive emotions.	- Blinding of participants, workshop leaders, and lab personnel. - Randomized controlled design. - Distinction of two meditation practices. - Larger sample size than past studies.
2	Le Nguyen Et al. [35]	- Blood samples collected at two lab visits. - Blinded study personnel. - DNA extracted using QIAamp DNA blood mini kit. - Telomere length measured via quantitative PCR.	- Insufficient sample size. - Variability introduced due to incomplete homework assignments. - Study groups unbalanced in baseline positive emotions.	- Long-term mindfulness meditation effects. - Control for lifestyle, gender, age, and ethnic group.
3	Mendioroz et al. [14]	- Blood obtained by venipuncture. - DNA fixed, processed, and analyzed using fluorescent PNA probe. - Methylation analysis of subtelomeric regions.	- Lower number of participants. - Limited statistical power. - Inability to establish causal relationships.	- Measurement of effects of long-term mindfulness meditation. - Control for various factors.
4	Hoge et al. [11]	- Blood obtained from venipuncture. - Genomic DNA extracted using QIAamp 96-spin DNA blood protocol. - Telomere length measured by quantitative real-time PCR.	- Small sample size. - Higher rates of depression in meditation group. - BMI differences. - Inability to distinguish effects of two meditation practices. - Lack of a measure of perceived stress.	- Control for various variables including BMI and depression. - Statistical analysis after controlling for covariates.
5	Alda et al. [15]	- High-throughput quantitative fluorescence in situ hybridization (HT-Q-FISH) for telomere length measurement.	- Small sample size. - Complex measurement processes and instruments. - Non-random assignment, unclear causality.	- Control group matched well with meditation group. - Inclusion of non-Caucasian participants for cross-cultural comparisons.
6	Carlson et al. [33]	- DNA extracted from blood samples. - High-throughput analysis using quantitative real-time PCR.	- Sample size insufficient for statistical power. - Longer time period needed for assessing mood/stress and TL relationship. - Missing data for intent-to-treat analysis.	- Use of trained clinicians for interventions.
7	Conklin et al. [13]	- Blood samples analyzed at UCSF lab. - Genomic DNA purified using QIAamp DNA Mini kits. - Telomere length measured by quantitative real-time PCR.	- Long-term effects not studied. - Variation in assay methodology and reference standards.	- Participants living on-site with controlled conditions. - Uniform diet for participants.
8	Duraimani et al. [36]	- Venipuncture blood draws for telomerase gene expression and leukocyte telomere length.	- Pilot study with a low sample size. - No data on absolute telomere length.	- Control and intervention groups well-matched.
9	Innes et al. [37]	- Blood samples collected before and after the intervention were processed to extract DNA using Qiagen's kit and stored at -20°C. Telomere length was measured using the Cawthon method, validated for high-throughput studies.	- Small sample size. - No diagnostic evaluation of cognitive status. - Lack of biomarker evaluation.	- Multiple assessment methods for cognitive function. - Additional post-intervention assessment 3 months later.
10	Keng et al. [38]	- Blood samples were collected before and after the intervention. - DNA extraction and telomere length measured by quantitative PCR.	- No blinding of researchers. - Shorter intervention period. - Lack of a no-intervention control group. - No assessment of other factors influencing telomere length. - No follow-up measurements.	- Well-matched interventional structures. - Large sample size.
11	Krishna et al. [39]	- Venous blood collected and stored. - Telomere length measured using quantitative PCR.	- Variation in yoga practice and diet among subjects.	- Statistically significant relationships found between LTL and dependent variables.
12	Lengacher et al. [40]	- Peripheral Blood Mononuclear cells collected at multiple time points. - Telomere length measured with quantitative rt-PCR.	- Small sample size.	- Measurement of both Telomerase Activity and Telomere Length. - Follow-up measurements 6 weeks post-intervention.
13	Tolahunase et al. [41]	- Peripheral blood samples analyzed for telomerase activity.	- Small sample size. - Psychological health variable kept constant.	- Significant relationships found between LTL and dependent variables.
14	Wang et al. [42]	- Leukocyte Telomere Length (LTL) measured twice with RT-PCR. - Genomic DNA extracted from frozen blood samples.	- Randomized group included participants with psychiatric disorders, not studied separately. - Limited statistical significance due to short study duration.	- Blinded investigators. - Use of multiple self-assessment questionnaires.

Discussion

Analysis of the articles included in this report revealed that randomized control trials were the most popular study design, with meditation being the most popular delivery of mindfulness intervention. The use of meditation or yoga was associated with significant increases in TL, while studies that utilized a dual-mindfulness protocol (meditation + yoga) did not observe any effects on TL. Studies that did not observe a significant effect on mood also did not observe any changes in TL [17,18] despite increased activity in TA.

Our hypothesis that randomized controlled trials would be the most effective design for studying mindfulness met the challenges of insufficient time and intensity of intervention and poor monitoring for compliance, while cross-sectional studies had the advantage of choosing participants who were long-term and regular practitioners of mindfulness. If we consider mindfulness as a skill that one may become more proficient in over time, this may explain why only one of the randomized controlled trials produced positive effects on TL. Long-term follow-up and serial telomere measurement might be beneficial in these cases. In this report, the shortest time frame in which benefits for TL were seen in novice mindfulness practitioners was six weeks [2], while that for experienced practitioners was three weeks [5]. Note that the latter was a controlled environment for 10 hours of daily meditation in the setting of a retreat. In order to produce robust and reproducible studies, achieving transparency of protocol and reasonable control of the confounding variables that can affect TL is crucial, and it is equally important to understand the skills and experience of the teacher delivering the intervention. This will allow their empirical findings to be translated into therapeutic interventions. As in pharmaceutical research, where the compound of interest and its therapeutic window must be clearly defined to provide a guideline for treatment, an analogous understanding of mindfulness and its “dosage” will be an essential point for study design.

Interestingly, the two trials that used a meditation program alone [2,5], as opposed to an MBSR, were the only two that observed significant positive effects on TL after the intervention. Similarly, the cross-sectional studies that we reviewed found an increased TL in participants who practiced a specific discipline of meditation or yoga [4,6,8,9]. Meditation and yoga are methods used to achieve both psychological and physical well-being; therefore, pre- and post-intervention analyses of psychological health are necessary to assess the validity of the intervention, as failure to produce such benefits may preclude somatic changes. MBSR differs from some mindfulness programs by offering instruction in various forms of mindfulness practices, providing participants with a broader toolkit of techniques. However, this approach may introduce an element of artificiality that doesn't always allow participants to fully benefit from fundamental mindfulness principles and adapt practices to their unique needs and preferences and researchers should consider this when designing future studies.

In the realm of non-randomized control trials, a single study was conducted involving the measurement of both telomerase activity and TL. This investigation yielded a significant positive effect on TL and negligible effects on telomerase activity. The study demonstrated control over environmental covariates; however, the long-term effects were not explored. Additionally, within the cross-sectional study design, four studies focused solely on assessing TL, all of which exhibited a significant positive impact on TL. Notably, three studies effectively controlled for multiple variables and accounted for covariates in their statistical analysis. Nonetheless, limitations were evident in the form of small sample sizes in two studies.

## Conclusions

In conclusion, the reviewed articles highlight a promising connection between mindfulness and TL, suggesting potential benefits for cellular aging and cognitive well-being. Future research should focus on meticulously designed mindfulness interventions that encompass diverse populations. To enhance clinical implications, customizing mindfulness practices for specific outcomes, extending intervention durations, and selecting target populations carefully are essential.

Furthermore, strengthening study methodologies with larger sample sizes, randomized control trials, and longitudinal assessments will bolster the evidence's reliability. These studies contribute to advancing our understanding of how mindfulness practices may influence cellular aging, offering promising prospects for future clinical applications.
